# Campbell title registrations to date—February 2023

**DOI:** 10.1002/cl2.1320

**Published:** 2023-03-19

Details of new titles for systematic reviews or evidence and gap maps that have been accepted by the Editor of a Campbell Coordinating Group are published in each issue of the journal. If you would like to receive a copy of the approved title registration form, please send an email to the Managing Editor of the relevant Coordinating Group.

**Ageing**

Effects of social prescribing for older adults: An evidence and gap map

Vivian Welch, Mojde Yaqubi, Elizabeth Tanjong Ghogomu, Kate Mulligan, Michelle Nelson, Marianne Saragosa, Caitlin Muhl, Sonia Hsiung, Cindy Yu, Paul Hebert

24 November 2022

**Business and Management**

Digital transformation in family firms: A systematic review

Tanapond Swanpitak, Aitthanatt Eitivipart

17 February 2023

**Children and Young Persons Wellbeing**

Impact of summer programmes on the outcomes of disadvantaged or at risk young people: A systematic review

Dan Muir, Becci Newton

27 January 2023

Socio‐cognitive determinants affecting insulin adherence/non‐adherence in late adolescents and young adults with type 1 diabetes: A systematic review

Hanan AlBurno, Francine Schneide, Hein de Vries, Dabia Al Mohannadi, Stefan Jongen, Liesbeth Mercken

18 January 2023

Frameworks of wellbeing for children and adolescents: A systematic review

Nicolas Aguilar‐Farias, Susana Cortés‐Morales, Sebastian Miranda‐Marquez, Marcelo Toledo‐Vargas, Jaime Carcamo‐Oyarzun, Andrea Cortinez‐O'Ryan, Alvaro Cerda‐Maureira, Natalia Quijada‐Mateluna, Karina Sandoval‐Aguilera, Jessica Ibarra, Constanza Andrade‐Torres, Teresa Balboa‐Castillo, Francisca Roman Mella

25 July 2022

**Disability**

The effect of nutritional supplement use in the management of symptoms in neurodevelopmental disorders: A systematic review

Bobbie Smith, Amanada Ludlow

13 February 2023

Scoping review on successful self‐employment evidence‐based frameworks used to promote self‐employment among persons with disabilities

Luther Lebogang Monareng, Shaheed Mogammad Soeker, Deshini Naidoo

16 January 2023

**Education**

The effectiveness of public‐private partnerships in education on educational access and quality of school education in low‐ and middle‐income countries: A systematic review

Sajid Ali, Sadia Muzaffar Bhutta, Sohail Ahmad, Aisha Naz Ansari, Gohar Shah, Yasir Maqbool Qadir, Afaq Ahmed

21 November 2033

The relations between school suspension, exclusion and medium‐ to long‐term student outcomes: A systematic review

Xinxin Zhu, Abby Lauren Pooley, Ingrid Obsuth, Harry Daniels, Ian Thompson, Aja Murray

24 January 2023

Universal school‐based programmes for improving social and emotional outcomes in children in the very early years (3‐4 years): A systematic review

Sarah Miller, Leonor Rodriguez, Daragh Bradshaw, Laura Dunne, Erin Early, Nicole Gleghorne, Jennifer Roberts

23 November 2022

School absenteeism among children and young people with special educational needs: A systematic review

Lara Stauvermann, Meike Rau, Vivian Meyer, Isabella Sasso, Michael Feldhaus, Karsten Speck

22 November 2022

**International Development**

The impact of public information on the electoral behaviour of citizens and the decision‐making of politicians in developing countries: A systematic review

Nan Chen, Kangle Guo, Yanfei Li, Lufang, Feng, Na Zhang, Jieyun li, Howard White, Xiuxia Li, Kehu Yang

09 February 2023

Risk factors of anemia in low‐ and middle‐income countries: A systematic review

Kavita Singh, Suchi Kapoor, Aakriti Gupta, Ashrita Saran, Howard White

15 November 2022

**Social Welfare**

The effectiveness of collaborative governance to improve the well‐being of the people those experiencing and at risk of homelessness: A systematic review

Tao Nian, Kuhu Yang, Xiuxia Li

09 February 2023

Individuation training for reducing implicit racial bias: A systematic review and meta‐analysis

Helena Garcez, Tiago O. Paiva, Catarina Botelho, Fernando Barbosa, Fernando Ferreira‐Santos,

10 January 2023

The effectiveness of Housing First for Canadians for those experiencing and at risk of homelessness: A systematic review

Gecheng Cui, Liujiao Cao, Xin Xng, Kehu Yang

21 December 2022

The effectiveness of behavioral interventions to promote smoking cessation amongst those experiencing and at risk of homelessness

Runjing Dai, Tiantian Feng, Hailiang Zhang, Na Zhao, Kuhu Yang, Jingchun Fan

19 December 2022

Neuropsychological assessment by videoconference in adults: A systematic review

Soraia Monteiro, Andreia Geraldo, Joana Pinto, Isabel Santos, Nick Defilippis, Fernando Ferreira‐Santos

23 November 2022

Health and social care interventions in the 85 years old and over population: An evidence and gap map

Rebecca Abbott, Morwenna Rogers, Alison Bethel, Rebecca Whear, Noreen Orr, Ruth Garside, Victoria Goodwin, Aseel Mahmoud, Ilianna Lourida, Debra Cheeseman, Jo Thompson Coon

11 November 2022

